# Multiple-Perspective Data-Driven Analysis of Online Health Communities

**DOI:** 10.3390/healthcare11202723

**Published:** 2023-10-12

**Authors:** Rana Alnashwan, Adrian O’Riordan, Humphrey Sorensen

**Affiliations:** 1 Department of Information Technology, College of Computer and Information Sciences, Princess Nourah bint Abdulrahman University, P.O. Box 84428, Riyadh 11671, Saudi Arabia; roalnashwan@pnu.edu.sa; 2 School of Computer Science and Information Technology, University College Cork, T12 K8AF Cork, Ireland; sorensen@cs.ucc.ie

**Keywords:** online health communities, Lyme disease, machine learning, sentiment analysis, content analysis, topic analysis

## Abstract

The growth of online health communities and socially generated health-related content has the potential to provide considerable value for patients and healthcare providers alike. For example, members of the public can acquire medical knowledge and interact with others online. However, the volume of information—and the consequent ‘noise’ associated with large data volumes—can create difficulties for users. In this paper, we present a data-driven approach to better understand these data from multiple stakeholder perspectives. We utilise three techniques—sentiment analysis, content analysis, and topic analysis—to analyse user-generated medical content related to Lyme disease. We use a supervised feature-based model to identify sentiments, content analysis to identify concepts that predominate, and latent Dirichlet allocation strategy as an unsupervised generative model to identify topics represented in the discourse. We validate that applying three different analytic methods highlights differing aspects of the information different stakeholders will be interested in based on the goals of different stakeholders, expert opinion, and comparison with patient information leaflets.

## 1. Introduction

There has been a large increase in the use of social media globally, including about health and medical matters [1]. In this work, we analyse the socially generated discourse in online medical communities, in particular, online forums discussing Lyme disease. We apply a data-driven approach to better understand the data from the perspective of different stakeholders, such as patients, medical professionals, and policymakers. This work falls within the remit of an emerging field of study, what Denecke calls Health Web Science [1] and what was previously referred to as Health 2.0 [2].

It has been recognised in the research literature that there are differences in how doctors and patients view conditions and treatments. Lenzi et al. write about a “potential misalignment” between the focuses of patients and doctors [3]. Other research has similarly identified misalignment between doctors and patients in terms of the perceptions of care, differences in medical knowledge, and misaligned emotional factors [4,5,6]. This study aims to elucidate these issues in the context of a sometimes-controversial medical condition.

Three approaches to data analysis—sentiment analysis, content analysis, and topic analysis—are employed. Each of the three approaches to data analysis has the potential to reveal different aspects of the data. Sentiment analysis is the computational analysis of people’s opinions, sentiments, emotions, and attitudes expressed towards a concept or entity [7]. Automatic content analysis of text documents relies on assessing high-frequency terms to deduce concepts from text [8]. Topic analysis is a method for identifying topics in a text collection and is based on a probabilistic model [9].

We intend to show how a large corpus of textual data extracted from health-related forums on the sometimes-controversial topic of Lyme disease can be analysed in different ways to reveal sentiments, concepts, and topics in the information space that could prove to be of interest to different stakeholders. In this way, a large diverse (and complex) information space can be viewed as a set of smaller, more coherent, and more comprehensible concepts and topics. We validate our findings by comparing them with the stakeholder goals, expert opinion, and patient information leaflets.

The remainder of this paper is organised as follows: Section 1.1 discusses the phenomenon of health-related social media and different stakeholder perspectives, Section 1.2 presents related work, and Section 1.3 our study context. Section 2 presents our methodology, with Section 3 detailing the three data-driven techniques to analyse the information. Section 4 presents our interpretation and validation and the analysis. Section 5 has conclusions and future work.

### 1.1. Health-Related Social Media and Stakeholder Perspectives

Health-related social media is increasingly being generated, shared, and analysed online [1]. The content is, primarily, narrative text about a medical topic that has been produced by concerned individuals, including patients, and sometimes doctors and other healthcare professionals. One of the most important benefits that social media offers is that health and medical information can be disseminated widely and accessed more conveniently across a broad range of the population, despite differentiation in age, education level, gender, and locality; this contrasts with traditional methods of communication and information dissemination. Although social media provides several benefits to health communities, there are issues regarding the quality and trustworthiness of the information and patient privacy [10].

There are many interested parties in healthcare, not just patients and doctors. These are often referred to as the stakeholders. These include patients; patients’ family members and caregivers; medical professionals, such as doctors, nurses, and other healthcare professionals; health services, hospitals, and clinics; and pharmaceutical companies, insurance companies, government, health regulators, research universities, and health charities. Sarashon-Kahn observes that consumers are “well ahead of other stakeholders in adopting social media in health” [11], suggesting that other stakeholders (physicians, health plan suppliers, and pharmacists) could use social media to gain knowledge to improve patient care.

In terms of the discussions on online forums, the principal stakeholders can be grouped into three perspectives: the individual perspective (patient, family, caregivers), the professional perspective (medics and other associated healthcare professionals), and the organisational perspective (health services, hospitals, policymakers, etc.).

#### 1.1.1. Individual Perspective

Individuals who participate on social media platforms include patients who may be suffering from a health condition and their families and carers. Social media with health-related content includes forums, blogs, social networking sites (in particular Twitter and Facebook), and video-sharing sites (in particular YouTube) [10]. Patients have become the biggest users of social media for health purposes.

Social media has had a considerable impact on the speed and nature of the interaction between individuals and between individuals and health professionals [10]. It has also become an increasingly important medium for those seeking medical information. A Pew Internet survey [12] reported that about 35% of US adults go online to identify a specific medical condition they or one of their relatives might have; these individuals are known as “online diagnosers”. In the report, 41% asserted that their diagnosis, condition, or disease was later confirmed by a medical professional. In addition, 26% of online users tend to search for others’ health experiences on social media. The concept of the expert patient is relevant here, referring to a patient with the knowledge and self-management skills to take ownership of their healthcare [13].

Online forums provide opportunities for participants to extend their reach beyond local general practitioners and hospitals [13]. Patients have the potential to use a variety of online health forums to ask questions, share their health knowledge (which is sometimes lacking in accuracy), provide and gain emotional support, learn from others, and express their concerns [1]. They often express and share their emotions, such as details about how they are feeling [14]. The forums facilitate the forming of vibrant online health communities in which thousands of communications on various conditions take place daily. Antheunis et al. carried out a questionnaire of 139 patients on their motives for using health-related social media [15]. They found that the main motive for patients to use Facebook was to “increase my knowledge on my disease” (39%) and to “express my emotions on my disease or health” (36%).

#### 1.1.2. Professional Perspective

Healthcare professionals include physicians, nurses, therapists, and public health officials. Healthcare professionals are becoming increasingly aware of the important role of patient involvement and patient preference in healthcare, which can now be seen in social media discourse and play a part in medical decision-making [1,16]. For example, compared with traditional surveys, online forums are a convenient way of obtaining a large amount of information from patients and their families to evaluate patients’ opinions and moods [17].

Physicians engage with social media for several reasons: to network, to gain insight into patient sentiment, to share information, and to give their time by answering patient questions online. Denecke reported on physicians using social media to enhance patient care [1]. Medical professionals can also gain valuable insights into how patients can support and influence each other. One controlled study demonstrated that written patient testimonials can have a significant impact on treatment choices [18].

#### 1.1.3. Organisational Perspective

Health organisations include hospitals, professional societies, clinics, health systems, and pharmaceutical companies. Organisations can benefit from social media to improve their image and visibility, market their services or products, seek funding, and create an online space in which to publish news about their activities [1]. Traditional methods of collecting opinions from the public and patients include Quality of Life assessments, interviews, and telephone surveys, but these are all time-consuming and limited in reach. Online technologies have become a fundamental part of public health surveillance [16]. Web information is now a valuable resource for public health agencies that depend on different sources for daily surveillance activities. Analysis of these online data complements the aforementioned approaches with additional sources of information. Online data analysis could enhance the potential for biomedical researchers to predict disease outbreaks more accurately [1]. It can also be used to ascertain public opinions on controversial public health issues. Policymakers in health organisations and government health departments are increasingly mining user-generated content on medical social media platforms to inform decision-making or assess service quality [19].

### 1.2. Related Work

There is existing work on stakeholder perspectives of medical social data. Denecke identifies three stakeholders who are invested in medical social media: patients or individuals, health professionals, and biomedical researchers, which can include organisations and researchers [1]. Furthermore, Deneke and Nejdl investigated the types of information available in medical social media, such as question-answering sites, blogs/forums, review sites, and wikis/encyclopaedias [20]. Their research shows that different types of content can be found in different knowledge sources. Patient-generated content on blogs/forums/answer portals mainly dealt with diseases and medications and had much affective content, whereas content generated by doctors and medical students in wikis/encyclopaedias was more informational and provided more information on anatomy/physiology and procedures.

Lu et al. carried out a study where they linked sentiment analysis and content analysis of medical social media to stakeholder perspectives [21]. They collected data related to lung cancer, diabetes, and breast cancer from MedHelp online forums. They identified three significant stakeholders: patients, caregivers, and specialists. The SentiWordNet lexicon was used to identify sentiment. Their content analysis consisted of feature extraction, probabilistic clustering, keyword extraction, and topic identification. Their feature extraction used word n-grams and UMLS terms. They recognised five significantly different health-related topics: symptoms, examinations, drugs, procedures, and complications. They found that the topics of interest and sentiment expression differed significantly among different stakeholders across the different disease forums.

Pattisapu et al. [22] proposed a persona classification analysis on online medical posts using a supervised approach. Their model was based on classifying online medical posts into one of various personas, including patients, caretakers, consultants, researchers, medical journalists, and pharmacists. They used a deep learning approach with CNN-LSTM and HANs neural architectures.

Sedereviciute and Valentini propose adapting a stakeholder analysis approach for social media that uses the Stakeholder Salience Model (SSM) and social network analysis [23]. They aim to identify and segment stakeholders to find “unknown” but important personas from social media that may be passive, such as lurkers, or active, such as influencers.

Lenzi et al. used topic analysis to identify the main complications, the frequently reported symptoms, and the common concerns of patients with diabetes [3]. They compare this with available quality of life (QoL) assessments obtained with standard methodologies, such as questionnaires to uncover differences in perception between patients and doctors. They found, for example, that issues reported to have a daily impact on patients were diet, glycaemic control, drugs, and clinical tests. These problems are not commonly considered in QoL assessments since they are not viewed by doctors as relating to severe limitations.

### 1.3. Data Subject Matter

Medical forums discussing Lyme disease are the focus of this study. Lyme disease (also called Lyme borreliosis) is a tick-borne infection caused by borrelia bacteria—*Borrelia burgdorferi* in North America but including other species in Europe and Asia [24]. Diagnosis is based on symptoms, a history of tick exposure, and possibly testing for specific antibodies in the blood. With the incidence increasing in many countries, it is becoming a serious public health concern worldwide. It is thus a disease that is topical and has varied symptoms. The purported existence of “chronic Lyme disease” is controversial and not recognised in mainstream, evidence-based medicine [25,26]. “[C]hronic Lyme disease … includes a broad array of illnesses or symptom complexes for which there is no reproducible or convincing scientific evidence of any relationship to *B. burgdorferi* infection” [25].

Members interact in online Lyme disease forums to gain the kind of social support that is important for people who may be suffering from the condition. Often, members post messages to support others, to clarify confusion regarding an infection, or simply to share their experiences, so that sufferers (or potential sufferers) can better deal with the disease. Researchers have found that patients who suffer from chronic conditions often build and maintain a long-term relationship with online health communities, which can establish a sense of belongingness [27]. In short, posts can come from a diverse population, can address diverse issues, and can have diverse aims.

Several medical forum sites (MedHelp, DailyStrength, PatientsLikeMe, Patient.info, and HealthBoards) have dedicated groups or communities focused on Lyme disease. Other online sources of information on Lyme disease include social networking sites (such as Facebook and Twitter), question-answering sites (such as Quora), and video-sharing sites (in particular, YouTube). As most of the content is user-generated, there are concerns as to the trustworthiness of some of the information [28]. An analysis of video content on YouTube about Lyme disease found that very little content is produced by academic or government agencies and the quality of the content could be improved [29].

## 2. Materials and Methods

Following the cross-industry standard process for the data mining (CRISP-DM) approach, our first step is business understanding to better understand the problem [30]. CRISP-DM is an open standard process model that gives a framework for data mining. It addresses such concerns as the steps to be carried out. Who generates and uses the data? To what end, for example, to learn, to assist in decision-making, to inform policy? We identified three different stakeholder perspectives: the individual or patient, the medical professional, and the organisational or policy viewpoint.

We posit that different analytic methods can identify and classify the concerns of a variety of stakeholders who might be interested in differing aspects of the information. We investigate three complementary analytics techniques—sentiment analysis, content analysis, and topic analysis—to leverage the best use of the data in an online health community.

Different methods of analysis focus on different aspects of the information collected. We first developed a model to identify the sentiment expressed by participants in individual posts to develop a classification model that can accurately identify sentiment across unstructured text messages. Next, we performed content analysis to obtain a general view of the medical content contained in the Lyme disease forums. Finally, we performed topic analysis to detect different topics represented by participants or patients in the health community utilising the latent Dirichlet allocation (LDA) model.

To validate the various sentiments, concepts, and topics identified, we relate these to the stakeholder goals. Further validation is furnished by consulting expert opinions and comparing the analysis with patient information leaflets.

### 2.1. Sentiment Analysis

Sentiment analysis is the process of analysing text to determine the emotional tone or affective states of a message [7]. Sentiment analysis is referred to by different names, such as opinion mining, attitude analysis, appraisal extraction, and sentiment mining. It has been applied across many domains, such as business, advertising, sports, and social science, to detect sentiment or opinions [7]. The principal approaches to sentiment analysis are the use of sentiment lexicons, machine learning techniques, or combinations of both. Sentiment can be assigned at different levels, sentences, aspects (features), or documents, or in the case of online forum posts, posts or threads. It is usually considered in terms of the assignment of positive, negative, and neutral sentiment. The degree or strength of the sentiment may also be calculated (typically in a numeric range such as −5 to +5). Emotion detection and multiclass sentiment analysis go beyond polarity classification to consider a set of afferent states (e.g., representing different emotions).

A characteristic of online medical forum discussion is the prevalence of affect or sentiment. Consequently, sentiment analysis has been widely applied to health-related social media [31], including to online medical forums [32,33,34,35,36,37,38,39].

We hypothesised that a binary sentiment classification (positive or negative) or polarity (degree to which positive or negative) would both be too limited and could not adequately address the range of sentiments expressed by forum participants due to the complex nature of medical posts. Most previous studies classify patient-authored content into positive, negative, and neutral, but this ignores a lot of the emotional richness in the text. Fewer studies have looked beyond sentiment polarity at emotion detection or have used multiple sentiment classes. For example, Nguyen et al. investigated emotion detection (anger, disgust, fear, joy, and sadness) in medical forum posts related to eye care to better understand patients’ perspectives and promote patient-centric care [38]. Wu et al. identified their own set of seven emotion labels or classes for text extracted from mental health discussions on psychpark, a virtual psychiatric clinic maintained by a group of volunteer professionals, including psychiatrists, psychologists, and other supporting team members in Taiwan [39]. Following this approach, we first developed a set of sentiment categories for posts from Lyme disease-related forums, and second, we performed automated sentiment classification concerning these categories.

The size of our data set (3000 posts) was compared with the sizes of the data sets used in all the cited work in sentiment analysis of health-related online forums. Data sets varied considerably in size from 1087 sentences to 138,161 posts. Note that while most of these studies used posts as the unit for sentiment assignment, some of these studies assigned sentiment as a sentence or thread (group of linked posts)-level task. The sizes of data sets collected in the related studies are as follows: 138,161 posts [21], not stated [22], 1087 sentences [32], 3500 posts [33], 1620 posts [34], 3600 posts [35], 1568 threads [36], 3515 posts [37], 46,381 posts [38], and 1711 posts [39].

#### 2.1.1. Domain-Dependent Categories Identification

As with other medical issues, Lyme disease is discussed on many online forums. We collected data from several user forums on MedHelp (https://www.medhelp.org/ (accessed on 19 March 2020) Lyme disease and Infectious Diseases communities) and DailyStrength (https://www.dailystrength.org/ (accessed on 19 March 2020) Lyme disease group) for one year.

The study collected freely available public data from Internet forums. In line with data privacy laws, UCC’s institutional Data Protection Policy requirements, and UCC policy for conducting Internet Research, all data were collected in accordance with the websites’ terms and conditions, held in full confidentiality, and anonymised. Names or any other information related to posters were not collected, nor were multiple posts by the same poster linked. There are no anticipated ethical issues with the work.

Posts reflect a broad cross-section of past, existing, and potential patients. Forum posts are usually long enough to convey sentiment [22]. This study adopted a bottom-up approach to develop and identify sentiment categories that are specific to Lyme disease forum discussion, the intention being to render these categories for a multi-class classification task.

**Figure 1:**
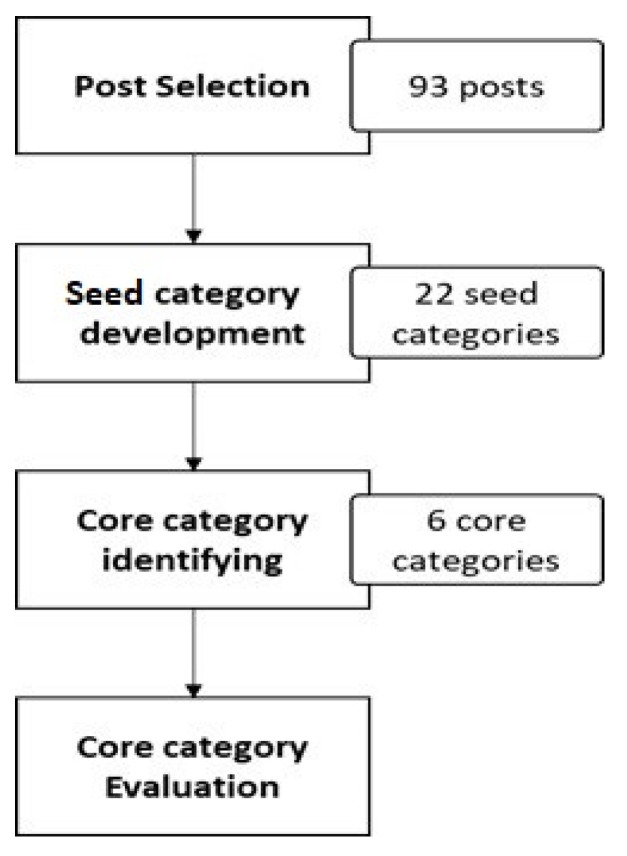
Processes for identifying the set of core sentiment categories. The chart shows how the initial 93 posts were reduced to six core categories in two steps. First, 22 seed categories were developed and these was further reduced to six. The core categories were then evaluated by MTurkers for coverage.

As shown in Figure 1, the process began with the selection of random posts. These posts varied in length: the shortest had 8 words, the longest had 730 words, and the average had 161 words.

In consultation with several medical professionals who have an interest in Lyme disease, each post was manually read and then categorised based on their observations. This facilitated the identification of a set of seed categories that emerged from the data. The seed categories started to be repeated; consequently, it was decided to terminate this step after manually classifying 93 posts, as no new categories were required. This initially resulted in 22 categories.

Some of the posts could represent more than one sentiment if we considered this as a sentence- or paragraph-level sentiment classification task. However, in this study, we placed each post into one meaningful seed category, a document-level classification task. The categories were then grouped and abstracted to a higher categorisation level, which we refer to as core categories. This process concluded after identifying six core categories presented in Table 1.

As a final step and to ensure the core categories were representative of the selected posts, we used a crowdsourcing service, the Mechanical Turk (MTurk), from Amazon. Although many of the posts were long enough to represent several sentiment categories, we requested that the MTurkers select the single predominant category for each post. For category evaluation purposes, we added a final category, “None of the above”, and asked the MTurkers to suggest and justify a new category based on their viewpoint. By adding this step, we could evaluate if the proposed categories were sufficient to cover all or most Lyme disease medical posts.

The final category set was determined by majority voting. The resulting annotations from the MTurkers were checked by the domain expert to verify their veracity. Of the total classification decisions, only 4.6% were “None of the above”. We took this to indicate that our proposed approach to identifying a comprehensive yet distinct set of sentiment categories was suitable.

#### 2.1.2. Sentiment Classification

The classification model used supervised learning on sets of features to classify posts. Our experiments consisted of three steps: the acquisition of a dataset, feature set generation, and classification. In the context of this work, while the classification is further validation of the six sentiment classes and gives a distribution of posts, the main output of the sentiment analysis for the stakeholder analysis is the sentiment classes given in Table 1.

We utilised the BeautifulSoup web scraper to collect approximately 3000 posts, all posts in the three forums under investigation going back one year.

The data were cleaned by performing the following steps. The text was tokenized (using the text analysis library devised by Microsoft Research) and case-folded, and certain classes of characters were removed (digits, special characters, email addresses, and URLs). Stop words were removed using the stop word list of 312 words provided by Azure Machine Learning. Lemmatization was applied (again using the Azure Machine Learning lemmatizer) to convert multiple related words to a single canonical form. All of the posts were in English. All of the posts were usable. The average length of a post was 161 words.

**Figure 2:**
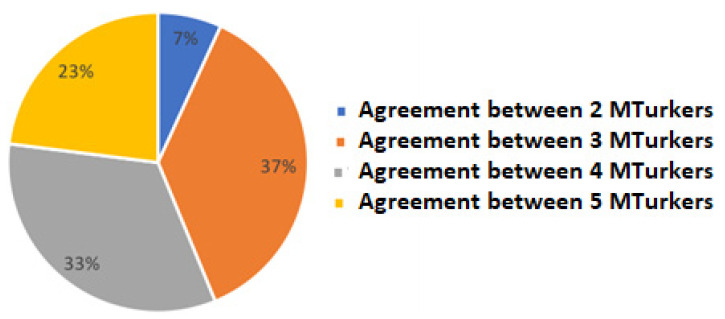
Level of agreement between the MTurkers in terms of assignment to a category.

We annotated posts using the services of Amazon’s Mechanical Turk. We asked five high-quality MTurkers (>97% approval rate) to choose the most dominant sentiment category for each post. Each post was assigned to its final category by a simple majority. Figure 2 shows the level of agreement of the data (after omitting the labeled posts with an uncertain sentiment category or when the “None of the above” category had been selected). The quality of the annotation was assessed using Fleiss’ kappa [40] for inter-annotator agreement. This annotation process reached a level of moderate agreement between annotations according to [41] (Fleiss’ kappa = 0.57).

For our experiment, a gold standard subset was created from the larger collection to achieve a high-quality annotated dataset with more agreement. The data selected were assigned to the same class by at least four annotators. This resulted in 1491 posts. The gold standard dataset score had a higher Fleiss’ kappa of 0.76, which indicates substantial agreement between the annotators [41]. Table 2 shows the final distribution of the gold standard labels among the sentiment classes.

#### 2.1.3. Feature Sets Generation

A key task in machine learning is to use appropriate features for the task at hand. We made use of three types of feature sets: content-free features, meta-level features, and content-specific features. Our baseline is feature hashing with n-grams. The feature-hashing model converts streams of words into sets of integers and vectors by creating a hashing dictionary of n-gram features calculated using the terms in the text.

Content-free features are lexical features that are either word-based or character-based. In total, we adopted 59 lexical features for each post based on Zheng et al. [42], collectively labelled LEX.

Meta-level features utilise several existing sentiment analysis lexicons which rely on a range of approaches to extract the sentiments from the text: supervised, unsupervised, and concept-based approaches. The outcome of these approaches is set in three dimensions: polarity, strength, and emotion. The meta-level features include polarity lexicons (POL), emotion lexicons (EMO), and a domain-specific lexicon (DOM). The polarity lexicons used were SentiWordNet, BingLiu, AFINN, NRC_Hashtags, Sentiment140 lexicon, SentiStrength, and a Sentiment140 method labelled as POL; the emotion lexicons were SenticNet and DepecheMood as EMO; and the domain-dependent lexicon was the HealthAffect lexicon as DOM.

Content-specific features included two additional features: a key phrase feature and n-gram feature extraction. Automated key phrase (KEY) extraction extracts topical words and phrases from the text. We transformed text into n-gram features (NG), which could then be used as a surrogate for the text by transforming frequently occurring text segments into a numeric vector representation. This feature generates a content-specific dictionary of frequency/inverse document frequency (TF-IDF) scores for each n-gram [43]. We used all unigrams and bigrams and, due to the high dimensionality of such a text feature, we excluded any n-gram that appeared in fewer than five posts or more than 80% of the posts. Finally, to select the most highly correlated n-grams, we used Chi-squared feature selection to choose 2000 desired features [44].

The experiments included the domain-independent (DI) feature set (LEX, POL, and EMO) and a domain-dependent (DD) feature set (DOM, KEY, and NG). A feature selection technique (chi-squared statistics) was again adopted to reduce the large number of features [22]. We selected the 500 most important features.

We tried out a variety of machine-learning algorithms for the classification model. Multiclass logistic regression (LR) and a multiclass neural network (NN) gave the best results in comparison with other approaches, such as a multiclass decision forest. We used an 80/20 data split, which is commonly used with randomised and stratified splitting: 80% of the data sample was used to train the model and the remaining 20% was used to test the performance evaluation.

### 2.2. Content Analysis

Content analysis is a research method that is used predominantly in the social sciences to analyse texts and written communications to indicate the presence of interesting, meaningful pieces of content [45,46,47]. An early definition is attributed to Berelson: “a research technique for the objective, systematic and quantitative description of the manifest content of communication” [47]. Content analysis has been employed to analyse varied media such as mass communications, historical and scholarly texts, and latterly social media, for purposes such as identifying key phrases or common themes or to attribute authorship and authenticity [46]. Content analysis may be performed with human input for the coding or labelling of content but is increasingly performed automatically by computers. A combination of various statistical and natural language processing techniques (such as term counts, code or label counts, term specificity, part-of-speech tagging, word embeddings, word-sense disambiguation, and named-entity recognition) can be used to extract meaningful content. Content analysis, although relying primarily on quantitative techniques, can be used in both quantitative and qualitative research studies. One purpose of content analysis is to describe the characteristics of a text or communication, the “what” of the message. That is the approach we take to analysing the medical forum data.

Content analysis was performed to gain insights into the concepts that appear in the online discussions. Statistical and natural language processing methods were employed to extract concepts from text. Swartz and Ungur provide an overview of the various content-based methods used to analyse social media data [8]. Content analysis has been previously applied in the medical domain [20,48,49,50,51].

The rule of thumb for content analysis is to consider high-frequency words to identify vocabulary of strong potential interest [45]. We extracted the most frequent concepts using text mining techniques, and to achieve a more semantically meaningful analysis, we mapped natural language to standard medical terminologies.

The gold standard dataset developed for sentiment classification was also used for content analysis. For the content analysis, we extracted key phrases to derive meaningful concepts. Key phrases are those that contain single noun words, compound nouns, or modifiers and nouns. The extracted key phrases were represented as n-gram features in a vector space representation using the widely known Term Frequency-Inverse Document Frequency (TF-IDF) weighting scheme [52]. Both word unigrams and bigrams are included in the term weighting. We applied count-based feature selection to exclude terms that appeared fewer than five times. This process resulted in 762 concepts in total.

The concepts identified were further reduced using domain-specific terminologies by mapping the terms to the Unified Medical Language System (UMLS) metathesaurus [53]. The UMLS is the largest repository of biomedical terminology in the world. It consists of knowledge that represents around 1.7 million biomedical concepts, classified under 133 semantic types. We used the US National Library of Medicine’s MetaMap software (MetaMap 2020 Release) [54] to map text to concepts in the UMLS. We then mapped concepts to a representative semantic type, resulting in a reduced total of 98 concepts.

Further reduction was then achieved by considering only those semantic types that appeared more than 10 times. This process resulted in identifying the 33 most frequent concepts or semantic types.

To represent and illustrate these concepts in a meaningful way, we calculated the similarity between the concepts using Word2Vec word embeddings [55]. Word2Vec is based on continuous bag-of-words and skip-gram architectures to derive a vector representation of concepts. In Word2Vec, the input is the text dataset, and the output is a vector space, in which words with similar meaning are proximate to one another. We used the Gensim implementation of Word2Vec [56]. We applied the pre-trained model (derived from a Google dataset of about 100 billion words) to generate a large word similarity matrix. We then used a hierarchical clustering function (agglomerative clustering with complete linkage [57]) to perform a cluster analysis to illustrate relationships in a conventional tree structure.

### 2.3. Topic Analysis

The final data analysis we carried out was topic analysis. Topic analysis of texts develops a topic model, a type of statistical or probabilistic model, for discovering the “topics” or themes that occur in that collection [9]. Early techniques included Latent Semantic Indexing, but the approaches most used today are latent Dirichlet allocation (LDA) models and extensions such as Pachinko allocation. Topic modelling is primarily applied to text data such as documents and social media posts but has been applied to other types of data such as image data and genetic information [9].

Topic analysis of social media has attracted the interest of many researchers interested in mining social media and the detection of topics of concern [58,59] and has been applied to the medical domain [60,61,62].

We used the popular unsupervised LDA model, a probabilistic model that depends on a hierarchy of components, the basic intuition being that each document (or post) can be assigned to multiple latent topics and each latent topic is distributed over a range of words [63]. We first applied the same pre-processing (tokenization, case-folding, stop word removal, and lemmatization) as we did in sentiment analysis. We employed the efficient Mallet implementation of the LDA model [64].

Various topic coherence matrices have been proposed to evaluate topic quality. For example, following extensive study of various measures, Röder et al. [65] proposed a topic coherence measure derived from a combination of known components, which resulted in a higher correlation to human ranking where the best performance compared well with other existing measures. We used Mallet’s built-in topic coherence measure (an implementation of the m_lc_(S_i_) measure as described in Röder et al. [65]) to identify the optimal number of topics from the data by testing the different values that represent the number of topics, from 2 to 30, using the --num-topics parameter in Mallet. This coherence metric measures whether the words in a topic tend to co-occur together by adding up a score for each distinct pair of top-ranked words. The score is the log of the probability that a document containing at least one instance of the higher-ranked word also contains at least one instance of the lower-ranked word with a smoothing parameter to avoid log zero errors. The number of iterations was left at the default value, which is 1000. We did not use any hyperparameter optimisation. Running the model multiple times, we found that average coherence increased until it reached seven topics but decreased after that, indicating that seven topics are optimal.

## 3. Results

### 3.1. Accuracy of Sentiment Analysis

To assess performance, we calculated the accuracy, precision, and recall. We computed precision and recall matrixes using two different approaches: micro-averaging and macro-averaging. Micro-averaging tends to be effective with the most frequent classes, while macro-averaging considers each class equally [43].

The experimental results for different feature sets and classification techniques are summarised in Table 3. The analysis was carried out in stepwise increments, whereby we progressively added features to the baseline. In most cases, the value of all five measures increased as various features were added. The results obtained from adding DI features outperform the baseline in the overall accuracy scoring. There is considerable improvement (more than 10% across all the performance measurements) after adding DD feature sets to the DI feature. Among the measurements for the five dimensions, feature selection (FS) performance showed significant improvement by filtering out irrelevant feature sets. There is consistency in the results for overall accuracy and micro- and macro-averaging. From Table 3, it can be observed that the multiclass neural network outperforms multiclass logistic regression.

#### Comparison with Expert Opinion

We also consulted expert opinion on the proposed sentiment analysis classification for Lyme disease posts. This was carried out through email discussion that included open-ended questions. A summary of the work and sentiment classes was shared with the participants. Of the 35 medical experts and practitioners with an interest in Lyme disease or infectious disease that we initially contacted, seven participated. They were from three countries (UK, US, and Ireland). The opinions are summarised in Table 4.

### 3.2. Content Analysis Results

**Figure 3:**
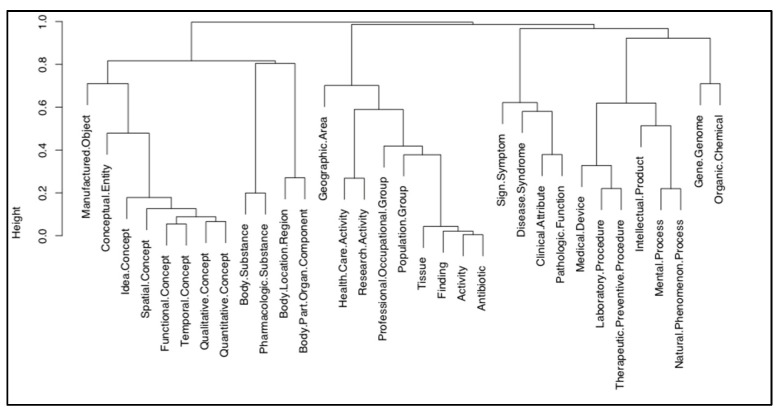
Cluster dendrogram (height is the depth of the tree, in steps of 0.2).

The results of the hierarchal clustering of the 33 most frequent topics (UMLS semantic types) are presented in the dendrogram in Figure 3.

### 3.3. Topic Analysis Results

In the LDA model, each distribution forms a collection of words representing seven different topics: initial symptoms after exposure, online patient communication, mental state, outline of the disease, treatment modalities, symptoms, and diagnostic testing. Table 5 illustrates the 20 keywords most associated with each topic.

#### Comparison with Patient Information Leaflets

We carried out a comparison of the topics identified with officially produced patient information leaflets on Lyme disease intended for the general public. These leaflets were all produced by reputable health organisations and public health agencies: The World Health Organization (WHO), the Centers for Disease Control and Prevention (CDC) in the US, the National Health Service (NHS), and Public Health England (PHE) in the UK, and Health Canada. The comparison is presented in Table 6.

## 4. Discussion

The various analysis techniques focus on different aspects of the information. A certain type of analysis can be more suitable for a particular perspective. Furthermore, the benefits can cut across groups and overlap. A single analysis technique might not be equally relevant to each group of patients, professionals, and organisations.

### 4.1. Interpretation of Sentiment Analysis

In the sentiment analysis, of the six core sentiment categories that were identified, three predominated: category 1, “Asking about treatment” (25% of posts); category 4, “Lyme symptoms confusion” (21%); and category 5, “Awareness and encouragement” (23%). This represents three of the major concerns that one might expect from sufferers and/or those worried about a controversial health topic: first, asking about possible treatments online; second, Lyme disease’s varied symptoms cause much confusion; and third, people turn to online medical forums for support and encouragement. The remaining less-prevalent core sentiment categories were category 2, “Depressed and frustrated” (8% of posts); category 3, “Lyme infection confusion” (16%); and category 6, “Seeking general information” (7%), which represent very negative sentiment, general confusion on the subject, and more neutral information-seeking, respectively.

The comparison with expert opinion revealed that, from the medical experts’ point of view, the sentiment analysis model could be of value from various perspectives, whether medical, patient, organisational, or service provision (see Table 4). One of the advantages stated by the physicians is that the analysis could be a way for patients to access accurate data that would meet their needs and concerns. One medic mentioned that it provides an effective and focused reading of online posts and can be used by medical experts as a means of ‘listening to patients’. According to one of the physicians who participated, “medicine is a healing art which is garnished by a veneer of science. Science is subject to changes and is constantly updated. The healing art remains and requires active listening to the patient”. According to another participant, “any number of services wanting to be could use this to generate FAQs covering these sort of themes”. This type of analysis could also assist medical researchers by identifying what variations people have experienced in their Lyme disease journey or what treatments have worked for them (e.g., those aimed at new or potentially diagnosed, those aimed at correcting misbeliefs about Lyme disease, etc.). One of the participants was, however, unable to see any value in conducting such an analysis; that expert did not have any confidence in the quality of Internet social data and regarded information posted on the Internet as potentially misleading.

### 4.2. Interpretation of Content Analysis

Figure 3 illustrates the concepts most discussed among the members of online health communities related to Lyme disease, as represented by the 33 domain-specific semantic types. Forum participants mention general medical concepts that are related to the Sign. Symptom and Disease.Syndrome semantic types. Concepts related to the Body.Location.Region and Body.Part.Organ.Component semantic types also appear with a high degree of frequency. Various concepts, such as area name, city, or county defined as the Geographic.Area semantic type were also mentioned; this may be because certain locations have a high risk or rate of infection, such as the northeast of the US.

The concept types identified are narrower in focus than the sentiment categories, probably because of the mapping to the UMLS medical thesaurus. The groupings are harder to interpret as well, as, for example, Laborary.Procedure and Mental.Proces are in closed related branches of the dendrogram.

### 4.3. Interpretation of Topic Analysis

The topic analysis identified seven significant health topics related to Lyme disease (see Table 5). Topic labels were interpreted by two medical experts based on the keywords extracted. According to these experts, the emergent topics found cover the general characteristics of the condition.

Topics #1 and #6 were closely related to symptoms, which is not surprising as the symptoms of Lyme disease can cause a lot of confusion. Furthermore, people tend to discuss symptoms a lot, as these become one of their greatest concerns. Some initial symptoms could indicate infection after being bitten by a tick, such as a rash. The duration and body area are important factors in identifying the initial symptoms, as found in topic #1 (initial symptoms after exposure). However, Lyme disease can include a combination of symptoms, and these can differ from person to person. Topic #6 highlighted symptoms such as pains in the legs, muscles, and joints, fatigue, and headaches.

Diagnostic testing was another major topic (#7), as laboratory blood testing (to identify antibodies to the bacteria) is the primary means to confirm a clinical diagnosis. However, some blood tests can produce a false-positive result, so patients tend to undergo another test, called the Western blot test, which was also found in the keywords.

Topic #5 addresses Lyme disease treatment modalities. From this topic, it can be observed that the only medication mentioned is antibiotics, as this is the standard treatment for Lyme disease. The posts related to the treatment topic contain abx as a keyword, which is a medical abbreviation for antibiotics.

Topic #3 encompasses patients’ mental states. Lyme disease can affect sufferers physically and mentally—patients can experience anxiety, depression, and a sense of loss, which can affect their lives, work, and sleep patterns. In response, the community surrounding Lyme disease patients often provides vital emotional support to help them cope with what they are facing. Therefore, support was one of the topics most discussed in the posts, with topic #2 highlighting online patient communication. This communication could motivate others to post their experiences and stories and to create and participate in groups such as forum communities.

There are many similarities between the six core sentiment categories and the seven topics that were identified by different data analysis methods. Sentiment category 1 and topic 5 relate to treatment. Sentiment category 2 and topic 3 relate to the patient’s mental state. Sentiment category 3 and topic 4 relate to questions about the disease. Sentiment category 4 and topics 1 and 6 relate to symptoms. Sentiment category 5 and topic 2 relate to the online communities. Sentiment category 6 and topic 4 represent more neutral information on the subject.

As can be seen from Table 6, the information contained in the leaflets and the topics identified in our analysis cover largely similar themes. An interesting observation is that there are two topics—Location and Prevention (A and B in Table 6)—that are mentioned in the leaflets but are not represented in our topic analysis; correspondingly, there are two social media topics—Online Patient Communication and Mental State—that do not have a corresponding representation in the leaflets. We would speculate that location and prevention would be more appropriate before any exposure and would be more likely found in information brochures; communication and mental state are of more concern to sufferers and would therefore be more topical in online forums. One could suggest that organisations and public health agencies should consider including a description of psychological effects in their leaflets because these are of considerable concern to those engaged in online social media discussion about Lyme disease.

### 4.4. Further Discussion: Benefits to Stakeholders

Individuals can be overwhelmed by the sheer volume of information available online on any given topic. The notion of information overload [66] and related concepts, such as information anxiety [67], are relevant here. If the outcomes of analyses (sentiment classes, concepts, and topics) were available to individuals, they could provide a useful map of the territory or information space that would allow users to navigate the information with more confidence and ease. Visualisations of sentiment, content, and topic analysis can greatly aid understanding. The SentiView application can be used to visualise sentiment [68]. In a health context, Zhang et al. present a visualisation tool for query terms used in a Web-based consumer health information system [69].

These analyses are a potentially valuable source for healthcare managers in transforming the data into information that can add value to health professionals’ work [1]. Sentiment information can alert professionals to particular areas causing patients distress. Content analysis can elucidate the knowledge held by patients, so practitioners, doctors, and organisations can better understand the concerns expressed online. Mapping the concepts that most concern individuals to domain-specific semantic types can also help address the terminology gap that exists between health professionals and health consumers. Topic analysis provides insights that would allow medical professionals and researchers to understand citizens’ most pressing concerns.

One scenario is that of a public health organisation wishing to collect specific data about a disease. They might want, for example, to be able to identify all the symptoms experienced by patients with Lyme disease. Health organisations could direct investment to those areas that are of most help to the community to optimise investment and cost-effectively generate more awareness. Moreover, this type of work could help identify misinformation regarding health issues and disseminate accurate health information to target communities.

### 4.5. Further Discussion: Generalisability of Sentiment Classes, Concepts, and Topics

The study focused on a single disease, Lyme disease. The question of whether the sentiments, concepts, and topics identified have wider applicability is worth consideration.

The six sentiment categories are sufficiently general that they could apply to other infectious diseases. And except for sentiment 3 (Lyme infection confusion), they could apply to other conditions, particularly of a chronic nature. But there could be others missing. Few existing studies apply a multi-class sentiment analysis approach to a medical condition, instead using a simpler binary or polarity classification. Here we review some of these few studies that have multiple sentiment categories.

Lu et al. [21], as discussed in Section 1, performed sentiment analysis and content analysis of data from online forums discussing breast cancer, lung cancer, and diabetes. They found five significant health-related concerns: symptoms, examinations, drugs, procedures, and complications. These were common across all three illnesses. However, there were differences in the distribution or prevalence of topics. Breast cancer patients were most concerned with examination topics (36% of posts), lung cancer patients were concerned most about symptoms (32% of posts), and diabetes patients were most concerned with drug topics (32% of posts). This study would suggest that the major categories identified are applicable across illnesses, but that different conditions have their unique profiles as indicated by the individual distributions of topics and sentiments.

Wu et al. [39], as discussed in Section 2.1, devised their own set of sentiment categories from mental health discussions. The seven categories identified were Depression, Mood, Drug, Insomnia, Social Anxiety, Schizophrenia, and OCD. Four of these are specific DSM-IV diagnoses, two relate to symptoms (Mood, Insomnia), and one to treatment (Drug).

Park and Ryu investigated the concerns of fibromyalgia patients from online posts [70]. Only a relatively small number (399) of patient testimonials were used. Note that fibromyalgia is a complex condition with varied symptoms and that causes patients a lot of confusion. Data analysis techniques used included parts-of-speech tagging, word frequency, and phrase identification. The concepts of most concern to patients included pain (the biggest concern), body parts (muscles, leg), symptoms (spasm, stiffness), and mental state (fatigue, depression).

While most of the 33 high-frequency concepts (UMLS semantic types) we identified by the content analysis were quite generic (e.g., Sign.Symptom, Laboratory.Procedure, Finding, Pharmacologic.Substance, Medical.Device HealtyCare.Activity, Population.Group), and none related directly to infectious diseases, some of the concepts (semantic types) indicate characteristics of an infectious disease like Lyme disease, such as the following semantic types: Antibiotic (treatment), Preventive.Procedure (prevention), Body.Location (diagnosis), Geographic.Area (location), and Mental.Process (patient distress).

He et al. analysed the data from online forums related to epilepsy to ascertain the major concepts [71]. They used part-of-speech (POS) tagging, phrase identification, and a mapping to UMLS Concept Unique Identifiers and UMLS semantic types. The top 10 concepts identified were (in order of frequency): seizure, epilepsy, physician, medicine, neurologist, Keppra, emotion, speak, sleep, and issue. The top UMLS semantic types in their study were: Sign.Symptom, Pharmacologic.Substance, Mental.Process, Finding, and Disease.Syndrome. Our study had these 5 semantic types as well among our 33 but differed considerably after their top 5.

From a review of existing studies that apply topic analysis techniques such as LDA to medical forum data [61,62], the topics identified tend to be specific to the condition or disease being discussed, so generalisation of topics to other conditions does not seem to be feasible.

## 5. Conclusions

We presented three methods of data analysis—sentiment analysis, content analysis, and topic analysis—that can be performed on text from discussions on Lyme disease. We give three perspectives of the data—the individual, the medical, and the organisation—and link these to the analysis. We show how these types of data-driven analyses can provide real benefits in a better understanding of the suffering of those with Lyme disease. More broadly, the work suggests that social media analysis is an influential source of knowledge for understanding the public’s attitude and knowledge of a disease.

### Scope for Future Work

Directions for future work are many. One direction would be to identify the different stakeholder groups in more detail. It may be possible to identify different types of individuals or patients: for example, to distinguish between chronic sufferers with a confirmed diagnosis, those with an acute issue, and those seeking information who do not know if they have a condition. The ‘expert patient’ is one who often provides a lot of information to others online and is an interesting phenomenon in its own right [13]. It may also be possible to separate the concerns of family members and carers from those of the patients themselves. The work of Lu et al. [21], previously described in Section 1.2, has explored this direction. They used text mining techniques to identify different stakeholders such as patients and caregivers. They found that patients were more likely to express their emotions than caregivers, although the distribution of topics was similar among caregivers and patients.

A consideration when performing content analysis is that the terms used in health-related content in social media can vary from the terminology used in the medical community [72]. This is sometimes referred to as patient language or Consumer Health Vocabulary. Many laypersons have difficulty with medical terminology or choose to use everyday or non-medical terms. In healthcare, patient language has been found to impact the quality of medical care [73]. Dirckx refers to the informal terms sometimes used by healthcare consumers as “patientese” [74]; these may contain abbreviations, slang, euphemisms, archaic medical terms, and unconventional use of grammar that differ from the current established medical terms used in medicine. As it stands, concept analysis is the least insightful of the three analyses we performed. Taking patient language into account could give a better set of concepts.

Unfortunately, inaccurate medical information can be promulgated on social media such as online forums, be it inaccurate diagnostic criteria or bogus or unproven treatments. This may be unintentional or malicious. It is useful for medical professionals to be aware of what possibly erroneous ideas are circulating in the community at any time to alert and inform patients. This was not further investigated in this work and remains a direction of future work. In a study by Cole et al. [75] of health information in online discussion forums, they found the information to be of “reasonably good quality”, although a small amount of information was assessed to be of poor quality. Inaccurate information could be identified if it diverged significantly from established medical opinion [76]. Various biases, be they socioeconomic, gender, age, race, etc., are inherent in any social network [77], including health forums [78,79], as not all sufferers can or will participate in online forums. Methods to reduce bias should lead to more accurate and informative analyses.

## Figures and Tables

**Table 1:** Description of the six core categories and their seed categories.

Category	Includes Seed Category
1. Asking about treatment	Asking about a specific treatment Asking about medication Medication is not helping and asking for an alternative
2. Depressed and frustrating	Desperate and depressed Disappointed with the community Loneliness Worried and confused about having new symptoms Disagreement
3. Lyme infection confusion	Being confused about having Lyme disease (if they have Lyme or not) Is it worthwhile pursuing a particular doctor?
4. Lyme symptoms confusion	Confusion if a specific disease (not Lyme disease) has these symptoms A patient diagnosed with Lyme but confused if symptoms relate to Lyme A patient who does not have Lyme, but is confused about the symptoms
5. Awareness and encouragement	Awareness and support Encouragement and support Providing general information Gratitude to his/her doctor
6. Seeking general information	Asking about advice Asking for information (from a doctor or specialist) Asking for information related to products (such as a Rife machine) Seeking test information Seeking a job

**Table 2:** Final distribution of the gold-standard data for the six categories.

Classification Category	Number of Posts	Percentage
1. Asking about treatment	377	25.3
2. Depressed and frustrating	118	7.9
3. Lyme infection confusion	235	15.8
4. Lyme symptoms confusion	317	21.3
5. Awareness and encouragement	335	22.5
6. Seeking general information	109	7.3
Total	1491	100

The table shows the six sentiment categories, the number of posts that were classified as that category, and in terms of the percentage.

**Table 3:** Sentiment analysis performance results for different feature sets.

		Baseline	DI Features	DI + DD Features	All Features +FS
Multiclass LR	Overall accuracy	0.607	0.638	0.715	0.732
	Micro-average precision	0.607	0.638	0.715	0.732
	Macro-average precision	0.628	0.676	0.705	0.74
	Micro-average recall	0.607	0.638	0.715	0.732
	Macro-average recall	0.539	0.567	0.66	0.678
Multiclass NN	Overall accuracy	0.55	0.631	0.721	0.745
	Micro-average precision	0.55	0.631	0.721	0.745
	Macro-average precision	0.516	0.594	0.7	0.73
	Micro-average recall	0.55	0.631	0.721	0.745
	Macro-average recall	0.516	0.595	0.671	0.705

Accuracy, precision, and recall for all feature set combinations. Legend for columns: Baseline is the baseline features we used (feature hashing with n-grams); DI is the domain-independent feature set and DD is the domain-dependent feature set; FS represents feature selection (chi-squared statistics). Legend for rows: Multiclass LR results are multiclass logistic regression and Multiclass NN the multiclass neural network classifier.

**Table 4:** Benefits of our model from physicians’ point of view.

Main Takeaway Points	Perspective		
	Medical	Patient	Organisation
A method of being able to “listen to the patient”	✓		
Could help practitioners with active learning as part of healing (constant) as the science changes (variable)	✓		
Assists understanding of the conflict in diagnosing Lyme disease	✓	✓	✓
Supports psychiatrists in providing a direct link for interpreting thought patterns to assist in therapies such as CBT	✓		
Plays a role in supporting experts to protect patients’ health	✓		
Helps understand the hysteria and chaos surrounding this infection	✓	✓	
Enhances patient-focused communication by providing relevant and needed information			✓
Can be a way of reaching the right data	✓	✓	✓
Allows observation of the variations in Lyme disease symptoms people have experienced or what treatments work			✓
There is no value to this work	_	_	_

Summary of the feedback from experts in the fields on how the data analysis can provide benefits. The benefits are listed in the rows. The relevance to each of three perspectives (individual, professional, and organisational) is given in the tick boxes. Note that a benefit can be relevant to multiple perspectives.

**Table 5:** Topics and keywords.

	Topic Name	Top 20 Words
1	Initial symptoms after exposure	Start, day, week, month, time, ago, rash, doctor, long, back, doxy ^1^, bite, bit, notice, year, experience, area, recently, idea, develop
2	Online patient communication	Post, disease, find, patient, support, information, share, great, group, site, call, read, article, info, make, story, cure, issue, news, forum
3	Mental state	Feel, bad, time, thing, make, sick, good, hard, work, lot, anxiety, give, life, today, back, night, live, part, year, lose
4	Outline of the disease	Disease, tick, chronic, find, doctor, treat, year, patient, treatment, infection, illness, medicine, people, include, diagnosis, bacteria, research, health, case, dr
5	Treatment modalities	Treatment, antibiotic, good, experience, llmd ^2^, hear, treat, work, read, med, year, eat, people, herx ^3^, put, supplement, abx ^4^, make, continue, give
6	Symptoms	Pain, symptom, feel, body, leg, muscle, joint, head, problem, eye, severe, hand, foot, leave, normal, fatigue, arm, feeling, back, headache
7	Diagnostic testing	Test, symptom, blood, positive, result, year, doctor, negative, diagnose, show, low, high, came_back, lyme, doc, band, lab, western_blot, question, genex

The table shows the seven topics identified in the topic analysis and the top 20 terms for each. ^1^ Doxy stands for Doxycycline, an antibacterial medication. ^2^ LLMD stands for Lyme Literate Medical Doctor. ^3^ A die-off reaction or Herxheimer reaction can occur when treating the Lyme germ or co-infections. ^4^ Abx is a medical abbreviation for antibiotics.

**Table 6:** Comparison of leaflet topics and topics identified on social media.

	Topics	WHO	CDC	NHS	PHE	Health Canada
1	Initial symptoms after exposure	✓	✓	✓	✓	✓
2	Online patient communication					
3	Mental state			Post-infectious Lyme disease mentioned		
4	Outline of the disease	✓	✓	✓	✓	✓
5	Treatment modalities	✓	✓	✓	✓	✓
6	Symptoms	✓	✓	✓	✓	✓
7	Diagnostic testing	✓	✓	✓	✓	✓
A	Location	✓	✓			✓
B	Prevention	✓	✓	Link provided	✓	✓

A tick indicates that the topic is represented in the patient information leaflet. Legend for columns: WHO is the World Health Organization, CDC is the Center for Disease Control and Prevention in the US, NHS is the National Health Service in the UK, and PHE is Public Health England in the UK. Legend for rows: one to seven signify the seven topics identified in the Lyme disease forum posts: initial symptoms after exposure, online patient communication, mental state, outline of the disease, treatment modalities, symptoms, and diagnostic testing, respectively. A and B are topics that appear in the leaflets but not the topic analysis.

## Data Availability

Not applicable.
